# Blood neurofilament light concentration at admittance: a potential prognostic marker in COVID-19

**DOI:** 10.1007/s00415-021-10517-6

**Published:** 2021-03-20

**Authors:** Anne Hege Aamodt, Einar August Høgestøl, Trine Haug Popperud, Jan Cato Holter, Anne Ma Dyrhol-Riise, Kristian Tonby, Birgitte Stiksrud, Else Quist-Paulsen, Tone Berge, Andreas Barratt-Due, Pål Aukrust, Lars Heggelund, Kaj Blennow, Henrik Zetterberg, Hanne Flinstad Harbo

**Affiliations:** 1 Department of Neurology, Oslo University Hospital, Oslo, Norway; 2 Institute of Clinical Medicine, University of Oslo, Oslo, Norway; 3 Department of Microbiology, Oslo University Hospital, Oslo, Norway; 4 Department of Infectious Diseases, Oslo University Hospital, Oslo, Norway; 5 Department of Mechanical, Electronic and Chemical Engineering, Oslo Metropolitan University, Oslo, Norway; 6 Department of Research, Innovation and Education, Oslo University Hospital, Oslo, Norway; 7 Division of Emergencies and Critical Care, Oslo University HospitalRikshospitalet, Oslo, Norway; 8 Department of Immunology, Oslo University Hospital, Oslo, Norway; 9 Research Institute of Internal Medicine, Oslo University Hospital, Oslo, Norway; 10 Department of Internal Medicine, Drammen Hospital, Vestre Viken Hospital Trust, Drammen, Norway; 11 Department of Clinical Science, University of Bergen, Bergen, Norway; 12 Department of Psychiatry and Neurochemistry, Institute of Neuroscience and Physiology, The Sahlgrenska Academy at the University of Gothenburg, Mölndal, Sweden; 13 Clinical Neurochemistry Laboratory, Sahlgrenska University Hospital, Mölndal, Sweden; 14 Department of Neurodegenerative Disease, UCL Institute of Neurology, London, UK; 15 UK Dementia Research Institute at UCL, London, UK

**Keywords:** SARS-CoV-2, COVID-19, Neurofilament light, Glial fibrillary acidic protein, Mortality

## Abstract

**Objective:**

To test the hypotheses that blood biomarkers for nervous system injury, serum concentrations of neurofilament light chain protein (NfL) and glial fibrillary acidic protein (GFAp) can serve as biomarkers for disease severity in COVID-19 patients.

**Methods:**

Forty-seven inpatients with confirmed COVID-19 had blood samples drawn on admission for assessing serum biomarkers of CNS injury by Single molecule array (Simoa), NfL and GFAp. Concentrations of NfL and GFAp were analyzed in relation to symptoms, clinical signs, inflammatory biomarkers and clinical outcomes. We used multivariate linear models to test for differences in biomarker concentrations in the subgroups, accounting for confounding effects.

**Results:**

In total, 21% (*n* = 10) of the patients were admitted to an intensive care unit, and the overall mortality rate was 13% (*n* = 6). Non-survivors had higher serum concentrations of NfL (*p* < 0.001) upon admission than patients who were discharged alive both in adjusted analyses (*p* = 2.6 × 10^–7^) and unadjusted analyses (*p* = 0.001). The concentrations of NfL in non-survivors increased over repeated measurements; whereas, the concentrations in survivors were stable. The GFAp concentration was also significantly higher in non-survivors than survivors (*p* = 0.02).

**Conclusion:**

Increased concentrations of NfL and GFAp in COVID-19 patients on admission may indicate increased mortality risk. Measurement of blood biomarkers for nervous system injury can be useful to detect and monitor CNS injury in COVID-19.

## Introduction

Emerging evidence suggest that respiratory syndrome coronavirus 2 (SARS-CoV-2) infection may affect the nervous system [1, 2]. Increasing numbers of patients with COVID-19 are reported to have neurologic, neuropsychological and neuropsychiatric symptoms and manifestations [1, 3–5]. Possible mechanisms for nervous system affection in COVID-19 have been suggested such as direct infection of the nervous system and inflammatory and autoimmune mechanisms [6–10], but the pathobiology is still incompletely known [11].

Early identification of central nervous system (CNS) manifestation may guide treatment algorithms and thereby improve clinical outcome. Meticulous neurological monitoring is important to assess the frequency and degree of nervous system affections in COVID-19 patients. Blood-based biomarkers for CNS injury, like neurofilament light chain protein (NfL) and Glial fibrillary acidic protein (GFAp), may be valuable tools for detection and monitoring manifestation during the acute phase of this infection. GFAp is an intermediate filament highly expressed in astrocytes and is increasingly used as a serum biomarker of astrocytic activation/injury [12]. NfL is a subunit of neurofilaments, which are cylindrical proteins exclusively located in the neuronal axons, that can be measured in blood as a marker of neuronal injury [13, 14]. In a recent study, neurochemical evidence of neuronal injury and glial activation in patients with moderate and severe COVID-19 infection was demonstrated by assessment of NfL and GFAp [15, 16]. However, more studies are required to clarify the nature of CNS injury and evaluate the usefulness of these biomarkers in COVID-19 patients.

The aim of this study was to explore the association between disease severity in COVID-19 patients and blood concentrations of NfL and GFAp.

## Methods

### Study population

This study includes 47 adult patients (≥ 18 years old) with COVID-19, as assessed by a positive SARS-CoV-2 polymerase chain reaction (PCR) test targeting the E-gene on oro- and nasopharyngeal specimens. The patients were consecutively recruited from Oslo University Hospital (*n* = 26) and Drammen Hospital, Vestre Viken Hospital Trust (*n* = 21) between March 6 and May 22 2020 to a clinical cohort study (Norwegian SARS-CoV-2 study; ClinicalTrials.gov, number NCT04381819). Clinical information including National Early Warning Score (NEWS) 2 and routine laboratory samples were for most cases collected within 48 h after hospitalization. Peripheral blood samples were drawn at inclusion, days 2–5 and days 7–10 during hospitalization and repeated later for patients who were hospitalized longer. Only patients with both clinical data and blood samples available for neurofilament analyses were included. Data were extracted from medical charts. Standardized neurological examinations were not performed. Using a modified version of the International Severe Acute Respiratory and emerging Infection Consortium (ISARIC)/World Health Organization (WHO) Clinical Characterization Protocol (CCP), clinical and routine data were abstracted from electronic medical records and deposited into an ISARIC (https://isaric.tghn.org) REDCap database (Research Electronic Data Capture, Vanderbilt University, TN, hosted by University of Oxford, UK).

### Sample processing and analyses of biomarkers

Serum samples were collected with 4 mL Vacuette^®^ (Greiner bio-one International) and processed within 1 h by centrifugation at 2000* g* for 10 min at room temperature. Serum aliquots were immediately stored at − 80 °C until analysis. Samples were thawed only once during the processing. Measurement of GFAp and NfL in serum samples were performed in the Clinical Neurochemistry Laboratory at the Sahlgrenska University Hospital, Sweden, by board-certified laboratory technicians blind to clinical data. We used commercially available single molecule array (Simoa) assays on an HD-X Analyzer (Human Neurology 4‐Plex A assay (N4PA advantage kit, 102153), as described by the manufacturer (Quanterix, Billerica, MA). A single batch of reagents was used; intra-assay coefficients of variation were below 10% for all analyses. The results of NfL and GFAp were compared with age-related reference limits established in house from 2000 healthy control individuals at the Clinical Neurochemistry Laboratory, Sahlgrenska University Hospital, Sweden (unpublished data).

### Statistical analysis

For statistical analyses, the R software with a common set of packages for the purpose was used [17]. Unique multivariate linear models were used to test for changes in the levels of all biomarkers on admission to address group differences in symptoms, clinical signs and outcomes. Age and gender were adjusted for in all linear models as confounding variables, while creatinine was included in all linear models for NfL since creatinine was a significant confounding factor in our dataset. To correlate between NfL and GFAp concentrations with levels of the other biomarkers, Pearson’s correlations were conducted. The biomarker data were logarithmic transformed to account for the lack of normal distribution. For the biomarkers with low resulting levels (between 0 and 1), a constant of 1 was added to avoid negative log transformed values. All tests were two sided and *p* values < 0.05 were considered significant.

### Ethical considerations

Informed consents were obtained from all patients or next-of-kin if patients were incapacitated of giving consent. The study was approved by the South-Eastern Norway Regional Health Authority (reference number: 106624).

### Sources of support

This study received funding from Oslo University Hospital and the Research Council of Norway Grant no 312780 and has received private donation from Vivaldi Invest A/S owned by Jon Stephenson von Tetzchner.

## Results

### Baseline characteristics

The mean age of the included 47 patients was 60.3 (SD 16.3, range 27–93) years and the male proportion was 72% (*n* = 34) (Table 1). On average, the patients had symptoms of COVID-19 infection for nine days (range 0–45) before hospitalization. The most common neurological symptoms among all patients were headache, ageusia, anosmia and confusion (Table 1). None of them had reported chronic neurological diseases. Mean NEWS2 was 4.4 (range 0–16) and significantly higher among non-survivors (Table 1 and Fig. 3). In total, 21% (*n* = 10) of the patients were admitted to an intensive care unit (ICU). Six patients (13%) died from COVID-19 during the hospital stay (Table 1).

**Table 1:** Characteristics of the COVID-19 cohort included in the study

	Baseline
(a) Characteristics	*n* = 47
Female % (*n*)	28% (13)
Age [mean (SD, range), years]	60.3 (16.3, 27–93)
Days from symptom onset until hospitalization (SD, range)	9.0 (7.7, 0–45)
Weight [mean (SD, range), kg]	80.1 (16.7, 54–110)
Height [(SD, range), mean cm]	173.8 (11.0, 160–195)
BMI [mean (SD, range)]	26.0 (4.6, 18.3–33.8)
Present and previous smoking % (*n*)	26 (12)
National Early Warning Score 2 (SD, range)	4.4. (3.8, 0–16)
Intensive care unit % (*n*)	21 (10)
(b) Symptoms and signs	
History of fever % (*n*)	89 (40)
Fever [mean, (SD, range), degrees Celsius]	37.9 (1.0, 35.9–39.8)
Cough % (*n*)	85 (34)
Fatigue % (*n*)	19 (8)
Anorexia % (*n*)	42 (8)
(c) Neurological symptoms	
Headache % (*n*)	37 (14)
Ageusia % (*n*)	21 (4)
Anosmia % (*n*)	16 (3)
Confusion % (*n*)	13 (6)
Seizures % (*n*)	2 (1)
Meningitis/encephalitis % (*n*)	5 (1)
Known dementia % (*n*)	6 (3)
Stroke % (*n*)	0 (0)
(d) Musculoskeletal symptoms	
Myalgia % (*n*)	68 (26)
Joint pain % (*n*)	26 (10)
(e) Biomarkers on admission	
Serum GFAp concentrations [mean (SD, range), pg/mL]	286.4 (221, 74–1212)
Above cut-off % (*n*)	48 (22)
Serum NfL concentrations [mean (SD, range), pg/mL]	33.7 (36.0, 5.8–174.4)
Above cut-off, %	30 (14)
CRP [mean (SD, range), mg/L]	97.4 (92.4, 0–400)
Ferritin [mean (SD, range), µg/L]	952 (747, 21–3465)
White blood cell count [mean (SD, range), × 10^9^/L]	6.5 (3.1, 2.6–18.0)
Procalcitonin [mean (median, SD, range), µg/L]	0.7 (0.1, 2.9, 0–16.3)
CK [mean (SD, range), U/L]	331.9 (733.4, 19–3572)
Creatinine [mean (SD, range), µmol/L]	95.8 (51.4, 55–281)
Neutrophil granulocyte count [mean (SD, range), × 10^9^/L]	4.8 (27, 1.3–11.3)

### Serum concentrations of NfL and GFAp in COVID-19 patients

**Fig. 1:**
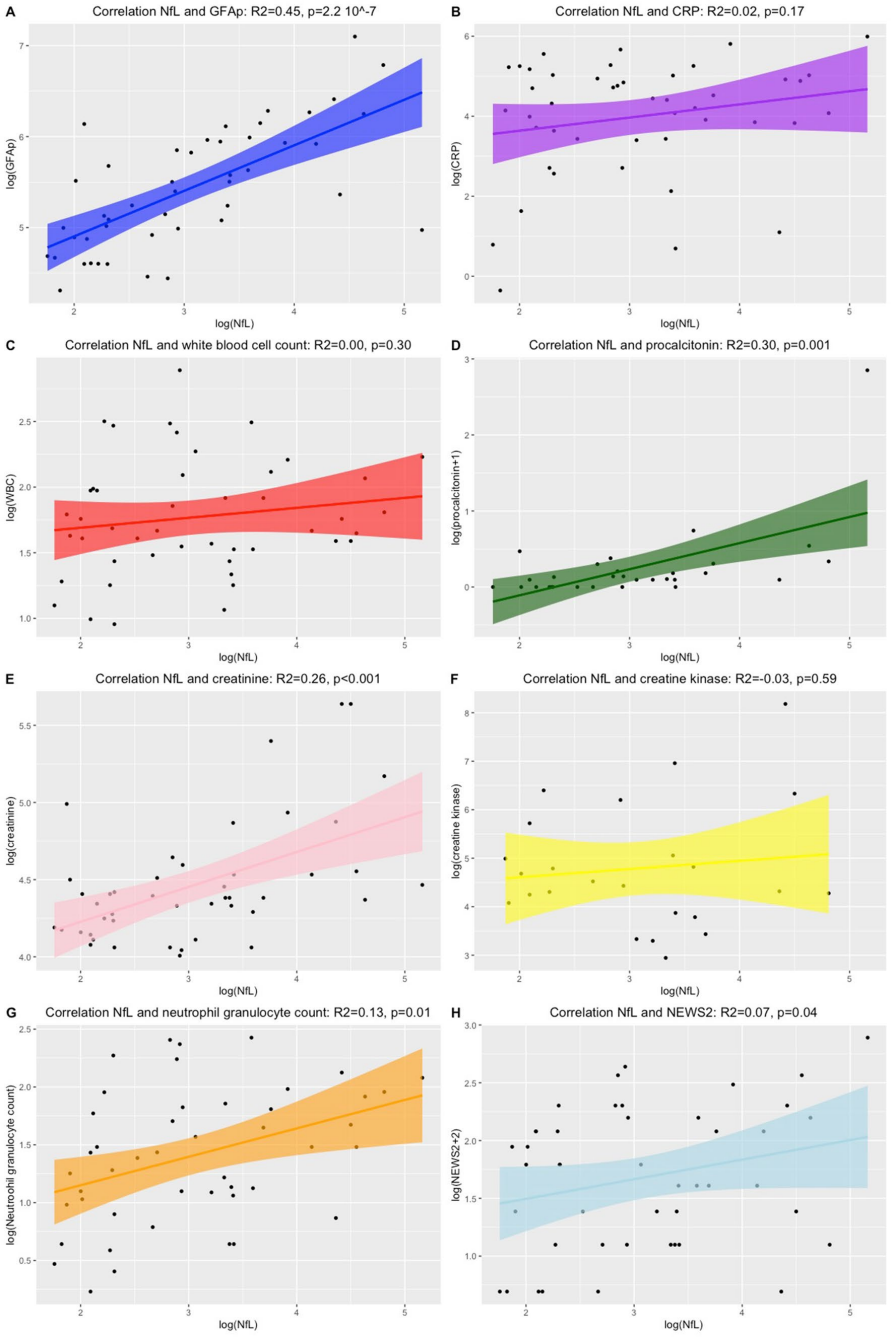
An overview of Pearson’s correlation between NfL concentrations and other biomarkers. Depicted are the correlations between NfL and GFAp concentrations (**a**), CRP (**b**), white blood cell count (**c**), procalcitonin (**d**), creatinine (**e**), creatine kinase (**f**), neutrophil granulocyte count (**g**) and National Early Warning Score (NEWS) 2 (**h**). Depicted are the logarithmic transformed values

**Fig. 2:**
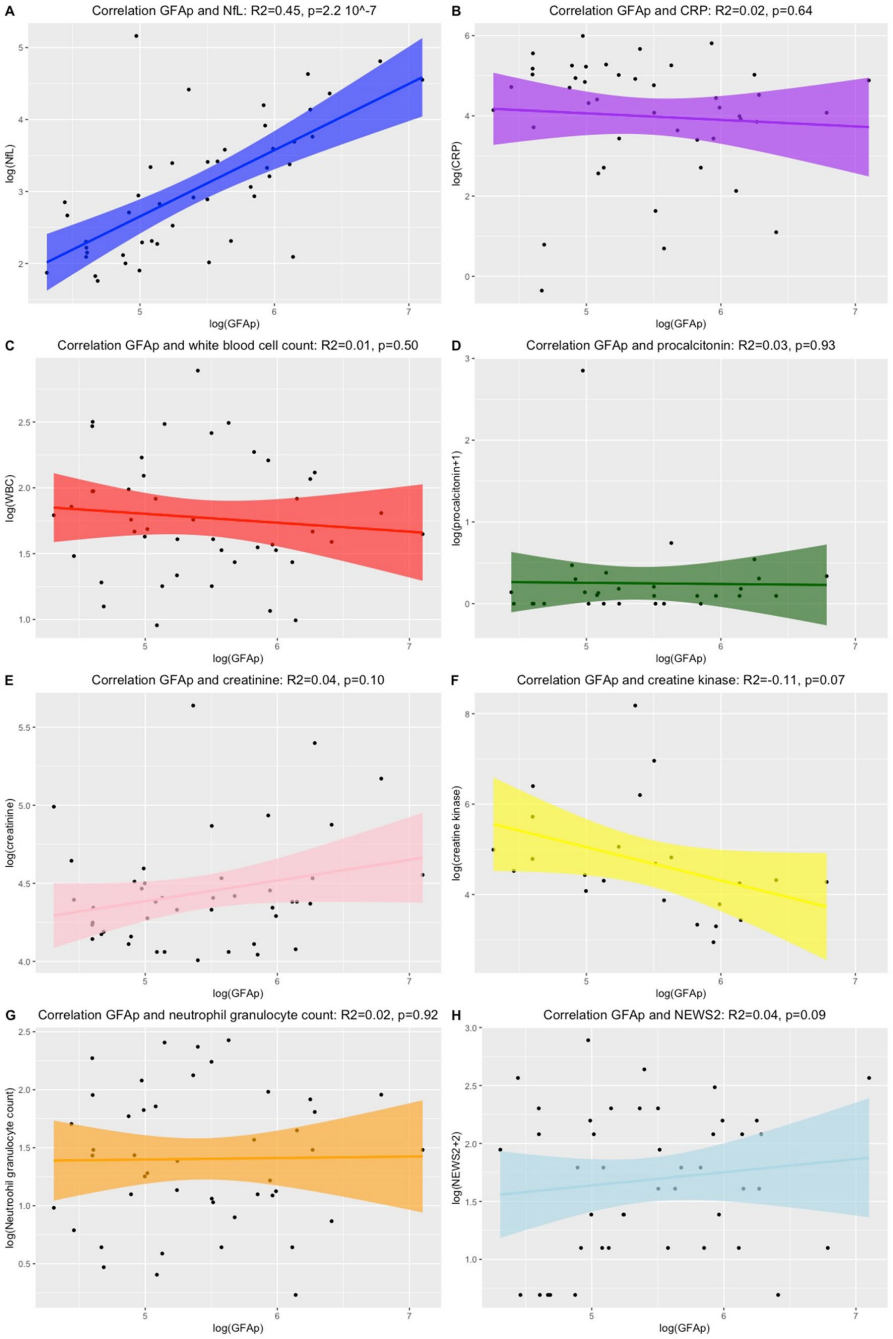
An overview of Pearson’s correlation between GFAp concentrations and other biomarkers. Depicted are the correlations between GFAp concentrations and NfL concentrations (**a**), CRP (**b**), white blood cell count (**c**), procalcitonin (**d**), creatinine (**e**), creatine kinase (**f**), neutrophil granulocyte count (**g**) and National Early Warning Score (NEWS) 2 (**h**). Depicted are the logarithmic transformed values

On admission, concentrations of NfL and GFAp above reference limits were measured in 30% (*n* = 14) and 48% (*n* = 22) of the COVID-19 patients, respectively (Table 1). Correlations between NfL concentration and GFAp (*p* = 2.2 × 10^–7^), procalcitonin (*p* = 0.001), creatinine (*p* < 0.001) and neutrophil granulocyte count (*p* = 0.01) as well NEWS2 score (*p* = 0.04) were found. No correlation was detected between NfL and GFAp with CRP, creatine kinase, ferritin or white blood cell count (Fig. 1). GFAp concentrations were only associated with NfL concentrations (Fig. 2, Table 2).

**Table 2:** Differences in NfL concentrations related to symptoms, treatment and outcome

Symptom	Linear regression, adjusting for age and creatinine (*R*^2^ = 0.26)		
	*t*	*R*^2^	*p*
Cough	3.16	0.38	**3.1 × 10**^**–3**^
Fatigue	2.50	0.34	**0.02**
Ventilatory support	1.40	0.28	0.17
Outcome—died	− 6.13	0.60	**2.6 × 10**^**–7**^
Anorexia	− 1.24	0.46	0.23
Confusion	− 0.95	0.26	0.35
Myalgia	3.59	0.42	**8.7 × 10**^**–4**^
Joint pain	2.86	0.37	**6.6 × 10**^**–3**^
Fever	− 0.37	0.24	0.72
Headache	1.73	0.29	0.09
Ageusia	− 1.54	0.48	0.14
Anosmia	0.06	0.41	0.95
Present and previous smoking	− 0.34	0.49	0.73
Intensive care unit	− 1.93	0.30	0.06

### Concentrations of NfL and GFAp in relation to clinical outcome

**Fig. 3:**
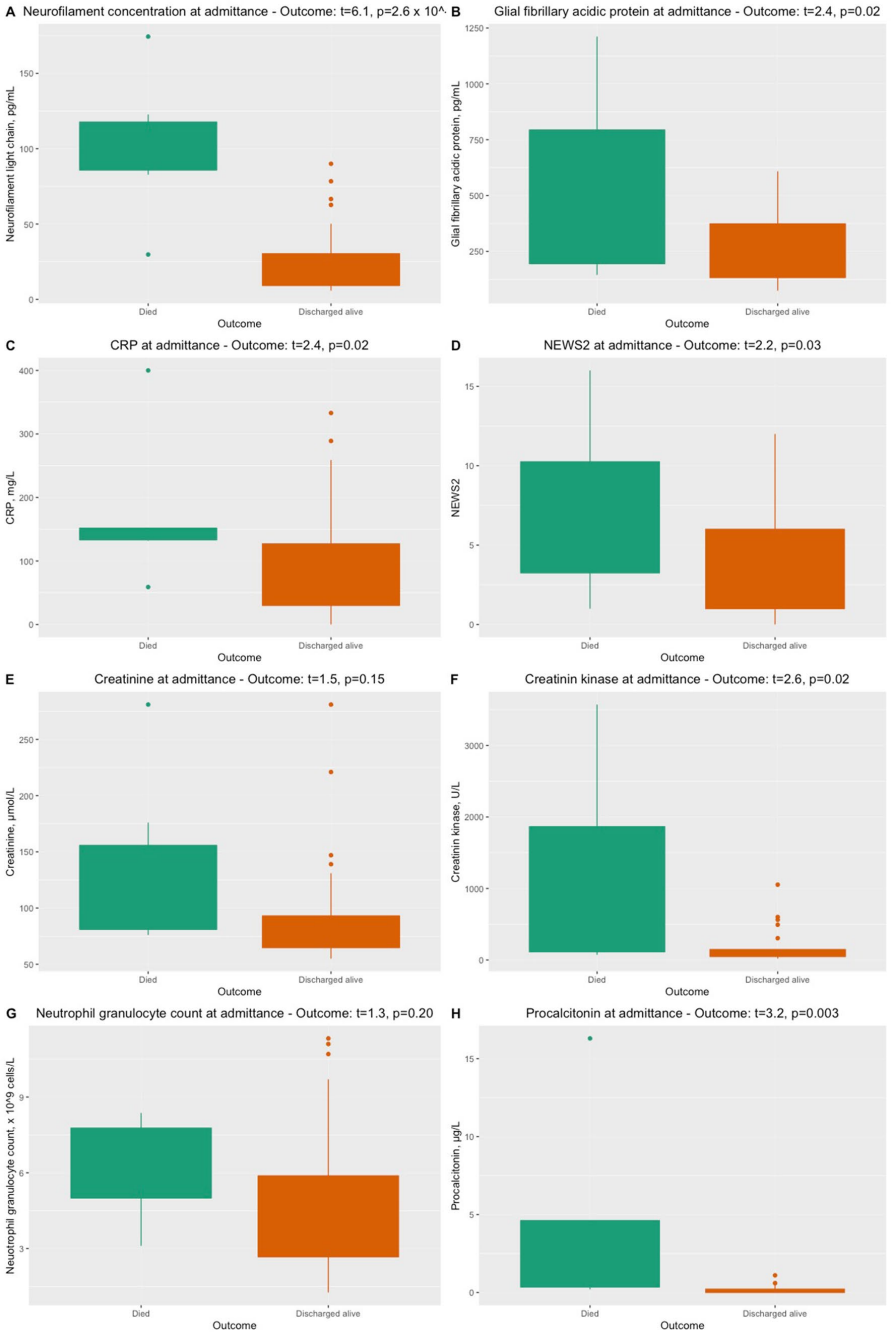
Levels of biomarkers among patients who died and who survived COVID-19 in this study. Statistical analyses performed with unique linear models adjusting for confounding effects. **a** NfL concentrations, **b** GFAp concentrations, **c** CRP, **d** National Early Warning Score (NEWS) 2, **e** creatinine, **f** creatine kinase, **g** neutrophil granulocyte count and **h** procalcitonin

Concentrations of NfL were significantly higher in non-survivors (*n* = 6) compared to survivors (*p* = 2.6 × 10^–7^) when adjusting for age and creatinine levels on admission (Fig. 3). Furthermore, higher concentrations of GFAp were significantly associated with a non-favorable disease outcome (*p* = 0.02) (Table 3). Significant differences among non-survivors compared to survivors were also observed in the adjusted linear models for the level of GFAp (*p* = 0.02), CRP (*p* = 0.02), creatine kinase (*p* = 0.02) and procalcitonin (*p* = 0.003) on admission but was not observed for the other biomarkers (creatinine or neutrophil granulocyte count) (Figs. 2, 3).

**Table 3:** Differences in GFAp concentrations related to symptoms, treatment and outcome

Symptom	Linear regression, adjusting for age (*R*^2^ = 0.40)		
	*t*	*R*^2^	*p*
Cough	0.57	0.37	0.58
Fatigue	1.81	0.43	0.08
Ventilatory support	− 0.95	0.40	0.35
Outcome—died	− 2.40	0.46	**0.02**
Anorexia	− 0.22	0.52	0.83
Confusion	− 1.44	0.42	0.16
Myalgia	1.86	0.44	0.07
Joint pain	1.78	0.43	0.08
Fever	0.37	0.39	0.72
Headache	− 0.26	0.39	0.80
Ageusia	0.00	0.52	1.00
Anosmia	0.70	0.53	0.49
Present and previous smoking	− 0.33	0.39	0.75
Intensive care unit	0.44	0.39	0.66

**Fig. 4:**
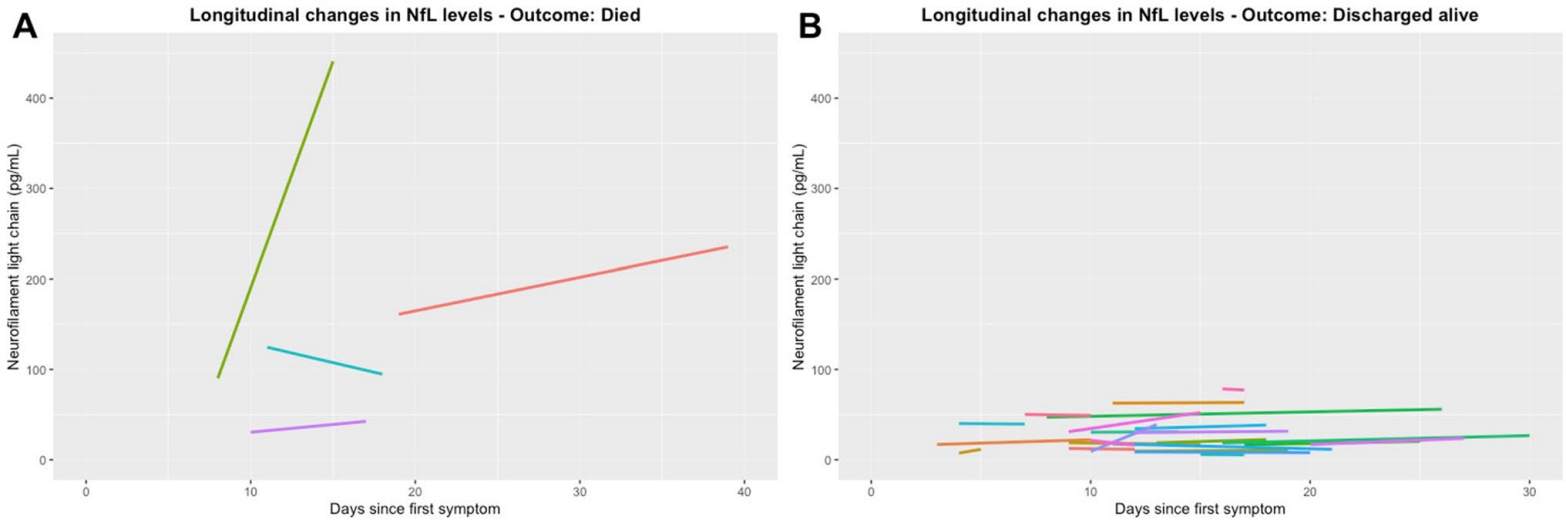
Longitudinal assessment of NfL concentrations among patients who died and who survived COVID-19 in this study. **a** Four subjects with longitudinal data who died. **b** An overview of the subjects who were discharged alive after hospitalization. Only subjects with longitudinal data are depicted

The longitudinal measurements of NfL concentration in patients available for this follow-up showed increased serum concentrations of NfL at hospital admittance and further increased concentrations during hospitalization in patients who died of COVID-19 (Fig. 4). The patients with the highest concentrations of NfL (> 120, max 464 pg/mL) had severe disease course resulting in death during hospitalization. They were all admitted with both respiratory and neurological symptoms (headache, dizziness) 4–7 days after disease onset. *x* The concentrations of NfL generally increased during the disease course in these subjects.

## Discussion

This pilot study indicates that increased concentrations of serum NfL in patients with COVID-19 may be a predictor of a severe disease course and increased mortality GFAp was also significantly associated with mortality. Increased NfL and GFAp concentration in patients with COVID-19 can be presumed to reflect affection of the nervous system. Although both the peripheral and central nervous system contain NfL, the correlation between CSF and blood is so strong that the majority of the NfL concentration must come from the CNS [18, 19]. Furthermore, GFAp is considered to be fairly specific to CNS [20]. The findings of high concentrations of NfL in non-survivors should be further studied in larger COVID-19 cohorts. Our findings are in line with another recent study of serum NfL concentrations in critically ill ICU patients where NfL concentrations were higher in COVID-19 patients than non-COVID-19 patients. Furthermore, higher NfL levels were associated with unfavorable short-term outcome [21].

Of other biomarkers available in this study, increased levels of procalcitonin were apparently associated with increased concentrations of NfL. However, this result is influenced by a few patients with very high measurements. Thus, the implications of these findings are not clear. Interestingly, NfL concentrations were not correlated with CRP and ferritin, often found to be associated hyperinflammation in COVID-19 patients, suggesting that the raised NfL concentrations merely reflect enhanced inflammation.

The association between clinical symptoms and NfL and GFAP in this study must be assessed with caution as the sample size was small. The patients with highest NfL values did all present with headache. Further neurological examination and evaluation was not available as they were all intubated shortly after admission. Furthermore, neuroimaging data were not available. The sample size was too small to draw other conclusions than fatal outcome. The lack of neurological examinations and of neuroimaging data does not allow to take in account the correlation with neurological involvement.

The identification of biomarkers in blood to assess nervous system manifestation will be important to monitor the severity of the disease and optimize treatment in COVID-19 patients. Measurement of NfL and GFAp in blood can be clinically useful methods to assess neurological affection in COVID-19, since this can easily be managed despite medical isolation procedures. Although NfL has been shown to be useful as diagnostic, prognostic and monitoring biomarker in a wide range of other neurological conditions [19, 22–24], more studies are needed to assess the applicability of NfL in COVID-19.

One could claim that the high concentrations of NfL could reflect medications used in ICU. However, a recent study of NfL and other blood biomarkers in patients undergoing inhalation general anesthesia showed a decrease in NfL concentrations after 5 h compared to baseline. This may suggest that the levels of NfL in COVID-19 patients treated in ICU might be even larger in magnitude but are masked by anesthesia-induced decreases [25].

The identification of biomarkers in blood to assess nervous system manifestation is important to monitor the severity of the disease and optimize treatment in COVID-19 patients. Measurement of NfL in blood can be a clinically useful tool to assess neurological affection in COVID-19. Although NfL has been shown to be useful as diagnostic, prognostic and monitoring biomarker in a wide range of other neurological conditions [19, 22–24], more studies are needed to assess the applicability of NfL in COVID-19.

In this pilot study, there are several limitations. First, the number of patients with full data sets available in this study was modest. Second, detailed and systematic neurological, neurophysiological and neuroradiological investigations were not possible to perform, since our patients were treated under medical isolation procedures at different units and several patients needed ventilatory support in ICUs. Thus, possible association between GFAp and NfL and specific CNS manifestations may have been undetected in this study. However, none of the patients with elevated NfL and GFAP levels had reported chronic neurological disorders. To expand our knowledge on the association between NfL and GFAp with neurological symptoms, we plan a follow-up study of COVID-19 patients up to a year after diagnosis including a systematic neurological assessment.

In conclusion, elevated concentrations of NfL and GFAp in COVID-19 patients seem to be potential prognostic markers in COVID-19. Further studies are essential to elucidate the pathogenesis and the clinical importance of how the COVID-19 disease affects the peripheral and CNS and how this can be measured and treated. Prospective neurologic and cognitive assessment of individuals with COVID-19 will also be crucial to understand the natural history of COVID-19 in the central nervous system and monitor for any long-term neurologic sequelae [26].

## Abbreviations

CNS: Central nervous system
SARS-CoV-2: Severe acute respiratory syndrome coronavirus 2
ACE-2: Angiotensin-converting enzyme 2
NfL: Neurofilament light protein
GFAp: Glial fibrillary acidic protein

## Declarations

### Informed consents

Informed consents were obtained from all patients or next-of-kin if patients were incapacitated of giving consent.
